# Altered amplitude of low-frequency fluctuation and functional connectivity in patients with acute unilateral vestibulopathy: a resting-state fMRI study

**DOI:** 10.3389/fneur.2024.1515262

**Published:** 2025-01-13

**Authors:** Zhengwei Chen, Liangqun Rong, Lijie Xiao, Jie Rao, Haiyan Liu, Tengfei Liu, Fei Chen, Jun Zhang, Lu Wang, Xi Li, Xiue Wei

**Affiliations:** ^1^Department of Neurology, Second Affiliated Hospital of Xuzhou Medical University, Xuzhou, Jiangsu, China; ^2^Department of Neurology, Lishui Central Hospital, Lishui, Zhejiang, China; ^3^Neurology Department, Third Affiliated Hospital of Shanghai University, Wenzhou Third Clinical Institute Affiliated to Wenzhou Medical University, Wenzhou People’s Hospital, Wenzhou, Zhejiang, China

**Keywords:** acute unilateral vestibulopathy, vestibular neuritis, fMRI, amplitude of low-frequency fluctuation, functional connectivity

## Abstract

**Objective:**

To investigate changes of brain functional activity in patients with acute unilateral vestibulopathy (AUVP) using functional magnetic resonance imaging (fMRI).

**Methods:**

We studied 32 AUVP patients and 30 healthy controls (HC) who received resting-state fMRI scanning. Methods of voxel-based amplitude of low-frequency fluctuation (ALFF) and seed-based functional connectivity (FC) were adopted to compare the difference in brain function between the two groups. In addition, we evaluated the associations between abnormal neuroimaging results and clinical data in AUVP patients.

**Results:**

Compared with HC, patients with AUVP showed lower ALFF in brain regions of bilateral insular, right precentral gyrus, left inferior frontal gyrus and right middle frontal gyrus, as well as higher ALFF in left cerebellar anterior lobe. Using these abnormal brain areas as seeds, we observed decreased FC between left insular and left precuneus in AUVP patients. Furthermore, AUVP patients showed increased FC between left insular and left supplementary motor area. Results of correlation analysis indicated that ALFF value (z-value) in left insular was negatively correlated with the canal paresis value (*p* = 0.005, *r* = −0.483), and the FC (z-value) between left insular and left precuneus was negatively correlated with dizziness handicap inventory score (*p* = 0.012, *r* = −0.438) in patients with AUVP.

**Conclusion:**

Patients with AUVP during acute period showed altered functional activity and connectivity in brain regions mainly involved in motor control and vestibular information processing. These changes in brain functional activity and connectivity were potentially attributed to decreased vestibular input resulting from unilateral peripheral vestibular impairment.

## Introduction

Acute unilateral vestibulopathy (AUVP), also previously known as vestibular neuritis (VN), is an acute vestibular syndrome. It mainly presents with acute or sub-acute vertigo and instability following acute impairment of vestibular function on one side, accompanied by nausea and vomiting, without hearing loss or central nervous system defects (1, 2). The annual incidence of AUVP has been reported to be 3.5–15.5 cases per 100,000 people (3, 4). AUVP is considered the sixth most common cause of vertigo/dizziness and the third most common cause of peripheral vestibular diseases second only to benign paroxysmal positional vertigo and Meniere’s disease (5). The usual age of onset of AUVP is 30–60 years old, and the peak age of onset is distributed between 40–50 years old (3, 4).

In clinical practice, AUVP patients often suffer from static and dynamic symptoms. At the same time, the body carries out static and dynamic compensation accordingly. The relief of static symptoms mainly depends on static compensation, which is believed to be related to the rebalance of electrical activity between the bilateral vestibular nuclei (6). Whereas, the full recovery of dynamic symptoms requires complex dynamic compensatory strategies that involve different brain regions (6). Recently, with the development of imaging techniques, an increasing number of studies have focused on the application of neuroimaging techniques to explore the brain metabolic, functional, and structural changes after unilateral peripheral vestibular damage (UPVD). Using 18F-fluorodeoxyglucose (18-FDG) positron emission tomography (PET) in VN patients during acute stage, Bense and colleagues reported increased regional cerebral glucose metabolism (rCGM) in multi-sensory vestibular cortical and sub-cortical regions, as well as decreased rCGM in visual, somatosensory and auditory cortices (7). Another 18-FDG PET study suggested that the mechanism of central compensation in AUVP was based on a shift of the dominant ascending vestibular input from the ipsilateral to the contralateral pathways (8).

In recent years, the application of functional magnetic resonance imaging (fMRI) and structural MRI provides new methods to explore the functional and structural alterations of the brain areas involved in patients with UPVD. In VN patients during chronic stage (more than 6 months’ post-onset), a task-state fMRI (visual and vestibular stimulation) study observed decreased activation in the primary visual cortex (V1) between patients and controls (9). The authors suggested that central compensation in patients with VN were partly mediated by adaptive mechanisms associated with the early visual cortex (9). In a resting-state fMRI study without any task or stimuli, patients with VN during acute phase displayed decreased functional activity in the contralateral intraparietal sulcus. Interestingly, the functional activity of this brain region increased 3 months later (10). These results indicated powerful compensatory cortical changes in resting-state activity in patients with VN (10). Another resting-state fMRI study indicated that the disturbed functional connectivity of default mode network provided some insights into mechanisms of central compensation in acute VN patients (11). A structural MRI study in acute VN patients using method of voxel-based morphometry (VBM) analysis demonstrated increased grey matter volume (GMV) in regions of vestibular cortex, bilateral hippocampus, visual cortex and the cerebellum, as well as decreased GMV in vermis and the prefrontal cortex (12). Another VBM study found increased GMV in acute VN patients’ multi-sensory vestibular cortices, cerebellum and the middle temporal area (motion-sensitive areas), as well as decreased GMV in the midline pontomedullary junction (13). In chronic stage VN patients (an average 2.5 years after onset of VN), a VBM study observed signal intensity increases for gray matter in the medial vestibular nuclei and the right gracile nucleus and for white matter in the area of the pontine commissural vestibular fibers (14).

Thus it can be seen, previous studies using neuroimaging methods such as 18-FDG PET, task-state fMRI, resting-state fMRI, and high-resolution T1-weighted images to investigate brain metabolic, functional, and structural changes in patients with AUVP have shown that multiple brain regions were altered following UPVD. Undeniably, these neuroimaging studies have significantly contributed to elucidating the complex central alterations associated with UPVD. However, the consistency and reproducibility of these studies are often lacking. This may be attributed to several factors, including relatively small sample sizes, clinical heterogeneity among participants, variations in imaging devices and scanning parameters, as well as diverse imaging analytical methods employed in previous studies. Furthermore, from a neuroimaging perspective, there is currently no specific theoretical model addressing central alterations in patients with AUVP. Therefore, using resting-state fMRI, we further explored the functional alterations in patients with AUVP. One notable feature of the current study is that patients with AUVP were diagnosed according to the diagnostic criteria established by the committee for the classification of vestibular disorders of the Bárány Society in 2022 (15). Unlike most previous research utilizing fMRI to investigate functional changes in patients with AUVP, the present study employed voxel-based amplitude of low-frequency fluctuation (ALFF) in conjunction with seed-based functional connectivity (FC) methods. Furthermore, we assessed the relationship between neuroimaging findings and the clinical characteristics of the patients. We hypothesized that patients with AUVP may exhibit altered functional activity and connectivity in brain regions associated with motor control and vestibular information processing.

## Materials and methods

### Subjects

We included 36 right-handed AUVP patients who were hospitalized in the Second Affiliated Hospital of Xuzhou Medical University. AUVP was diagnosed based on the criteria published by the Bárány Society in 2022 (15). All patients received routine neurological and neuro-otological evaluation, including demographic information, history of present illness, past medical history, neurological and vertigo bedside examination, conventional MRI (T1WI + T2WI + FLAIR+DWI), vestibular laboratory examinations (videonystagmograph (VNG), video head impulse test (vHIT), vestibular-evoked myogenic potentials (VEMPs) and rotatory chair test), audiogram and otoscopy. In addition, each patient underwent resting-state fMRI, as well as assessments of Montreal Cognitive Assessment scale (MoCA), Hamilton Anxiety Scale (HAMA), Hamilton Depression Scale (HAMD), Vertigo Visual Analogue Scale (VVAS; 0, no vertigo; 10, worst vertigo) and Dizziness handicap inventory (DHI). Patients with neurological, neuro-otological, mental or systemic diseases were excluded. Patients with a history of AUVP, drug or alcohol abuse were excluded. Patients were not allowed to take vestibular function inhibitors within 2 days prior to assessments of vestibular function and fMRI. No patient showed evidence for central neurological signs and symptoms. No patient had hearing impairment, tinnitus or otalgia. Patients with mild vestibular dysfunctions unrelated to AUVP were not included in the present study. To control for laterality, all patients suffered damage to the right side. All patients showed spontaneous peripheral vestibular nystagmus on VNG. Vestibular function examinations showed that all patients had reduced vestibulo-ocular reflex (VOR) function on the opposite direction of the fast phase of the spontaneous nystagmus (VOR gain less than 0.7 with saccades for vHIT, or canal paresis more than 25% for rotatory chair test).

Thirty-two right-handed volunteers served as healthy controls (HC). They had no history of dizziness or vertigo. None of them had a history of neurological, neuro-otological, psychological, or systemic disorders. Volunteers with history of drug or alcohol abuse were excluded. All HC received conventional MRI and resting-state fMRI, as well as assessments of MoCA, HAMA and HAMD. This study was approved by the Ethics Committee of the Second Affiliated Hospital of Xuzhou Medical University. All participants signed informed consent before entering the study.

### Imaging acquisition

The fMRI examinations were carried out on a 3.0 T GE MRI scanner (GE Medical Systems) at the medical image center of the Second Affiliated Hospital of Xuzhou Medical University. Participants were instructed to close their eyes, lie flat and still and remain awake during scanning. We adopted a 3D-BRAVO sequence to acquire T1-weighted images with the following scanning parameters: repetition time (TR) = 2,500 ms, echo time (TE) = 3.5 ms, flip angle (FA) = 8°, matrix size =256 × 256, field of view (FOV) = 250 mm × 250 mm, thickness/gap = 1/0 mm, slice number = 156. In addition, we used a fast field echo-planar imaging (EPI) sequence to obtain the fMRI images (TR = 2000 ms, TE = 30 ms, FA = 90°, FOV = 200 × 200 mm, matrix size = 64 × 64, thickness/gap = 3.6/0 mm, the total scan time was 7 min).

### The fMRI data preprocessing

The fMRI data were processed using CONN toolbox (version 18b)^1^ and Statistical Parametric Mapping 12 (SPM12)^2^ working on MATLAB R2023a platform (MathWorks, Inc., Natick, MA, USA). After images format conversion (DICOM to NIFTI), the first 10 time points were removed, the remaining 200 functional volumes of each subject were preprocessed with the following procedures: (1) to reduce the difference in the acquisition time of each layer during scanning, slice-timing correction was performed; (2) realignment (subject motion estimation and correction): the threshold of subject-motion was 2 mm; (3) functional outlier detection: Artifact Detection Tools (ART)-based identification of outlier scans for scrubbing was performed to remove the aberrant time points (a global-signal z-value threshold of 9); (4) to reduce the inconsistency of structural center caused by manual positioning by MR technicians during each scan, structural center to (0, 0, 0) coordinates was performed; (5) segmentation and normalization (DARTEL): the DARTEL method was adopted to register the structural space and functional space to realize the conversion from a single space to the standard Montreal Neurological Institute (MNI) space, and then all images were resampled to 3 × 3 × 3 mm^3^; (6) smoothing was performed based on a Gaussian kernel of 6 mm full-width at half maximum to improve the normality of the images; (7) band-pass filter: the spatially normalized images were passed through band-pass filter (0.01–0.08 Hz); (8) linear regression of confounding effects: white matter, cerebrospinal fluid, global mean signal and motion realignment parameters were regressed out. Four patients and two HC were excluded due to large head-motion or poor normalization. Finally, 32 patients with AUVP and 30 HC were included in the following analysis.

### ALFF calculation

The power spectrum was obtained by transforming the filtered time series into frequency-domain data using the fast Fourier transform (FFT). The mean square root of the power spectrum in the range of 0.01 to 0.08 Hz was calculated as the ALFF value. Then the ALFF value for each subject was converted to a z-score for further between-groups comparison.

### FC calculation

A seed-based method was used to calculate the FC between seeds and the rest of the brain voxels. Six brain regions that showed significant group differences in ALFF values were chosen as seeds. Seed 1: left insular [coordinates (−38, −7, 9)]; Seed 2: right precentral gyrus (PreCG) [coordinates (27, −3, 45)]; Seed 3: left inferior frontal gyrus (IFG) [coordinates (−51, 12, 9)]; Seed 4: right insular [coordinates (45, −9, 3)]; Seed 5: right middle frontal gyrus (MFG) [coordinates (51, 48, 3)]; Seed 6: left cerebellar anterior lobe (CAL) [coordinates (−20, −87, −24)]. The seed area was made with the above six spatial coordinates and a radius of 5 mm. We extracted the mean time series for each seed from the smoothed images. Then, Pearson correlation coefficients (r) between each seed and the remaining of the brain voxels were calculated and converted to z-scores by Fisher r-to-z transformation.

### Statistical analysis

#### Analysis of demography and clinical characteristics

The Statistical Package for the Social Sciences (SPSS Institute Inc., Chicago, IL, USA, v22.0) for Windows was used to analyse data of demography and clinical characteristics. We adopted two-sample *t*-tests to compare the group differences in age, years of education, score of HAMA, HADM, and MoCA. The chi-square test was applied for the analysis of gender. The significance level was set to *p* < 0.05.

#### Analysis of differences in ALFF and FC

Differences in ALFF and FC between patients with AUVP and HC were evaluated using two-sample *t*-tests, with age, gender, educational years, scores of HAMA, HADM, and MoCA as covariates considering their confounding effects. The significance threshold was set at voxel-level threshold (*p* < 0.001) and cluster-level threshold (*p* < 0.05, false discovery rate (FDR) corrected, two-tailed).

### Correlation analysis

For FC and ALFF (z-values) showing significant differences between the two groups, Pearson’s partial correlation analysis was used to examine the relationship between neuroimaging results and clinical features in patients with AUVP, controlling for age, gender, year of education, scores of MoCA, HAMA and HAMD. A significant correlation was defined as a *p* < 0.05.

## Results

### Demographic and clinical features

The demographic and clinical characteristics of the two groups were summarized in Table 1. There was no significant difference in age, gender, education level, scores of HAMA, HAMD and MoCA between patients with AUVP and HC (all *p* > 0.05). All AUVP patients we enrolled showed normal cognitive function, with no obvious symptoms of anxiety or depression. Patients suffered from moderate to severe vertigo which resulted in moderate dizziness handicap in this study. The duration of AUVP ranged from 1 to 6 days (2.8 ± 1.4 days). 9 (28.1%) patients had a clear history of upper respiratory infection before onset. All patients (32, 100%) had right peripheral vestibular lesions. All patients (32, 100%) showed peripheral spontaneous nystagmus, abnormal vHIT and canal paresis (CP, 60.6 ± 20.0%). Ocular VEMP (oVEMP) was abnormal in 25 cases (78.1%) and cervical VEMP (cVEMP) was abnormal in 10 cases (31.2%).

**Table 1:** Demographic information and clinical characteristics of the participants.

	AUVP (*n* = 32)	HC (*n* = 30)	*p*-value
Age (years)	44.6 ± 11.5	42.0 ± 6.7	0.286
Sex (male/female)	13/19	15/15	0.459
Education (years)	14.9 ± 2.9	16.2 ± 3.5	0.125
MoCA scores	27.1 ± 1.7	27.6 ± 1.4	0.153
HAMA scores	8.3 ± 2.3	7.4 ± 2.0	0.084
HAMD scores	9.7 ± 2.8	8.7 ± 2.2	0.115
VVAS scores	6.7 ± 2.0	N/A	N/A
DHI scores	59.7 ± 18.3	N/A	N/A
Duration of AUVP (days)	2.8 ± 1.4	N/A	N/A
CP (%)	66.7 ± 18.7%	N/A	N/A
Spn-SPV (°/s)	13.6 ± 11.9°/s	N/A	N/A

AUVP, Acute unilateral vestibulopathy; HC, healthy control; MoCA, Montreal cognitive assessment scale; HAMA, Hamilton anxiety scale; HAMD, Hamilton depression scale; VVAS, Vertigo visual analogue scale; DHI, Dizziness handicap inventory; CP, Canal paresis; Spn-SPV, Slow-phase velocity of spontaneous nystagmus; N/A, Not applicable.

### ALFF differences between groups

**Figure 1:**
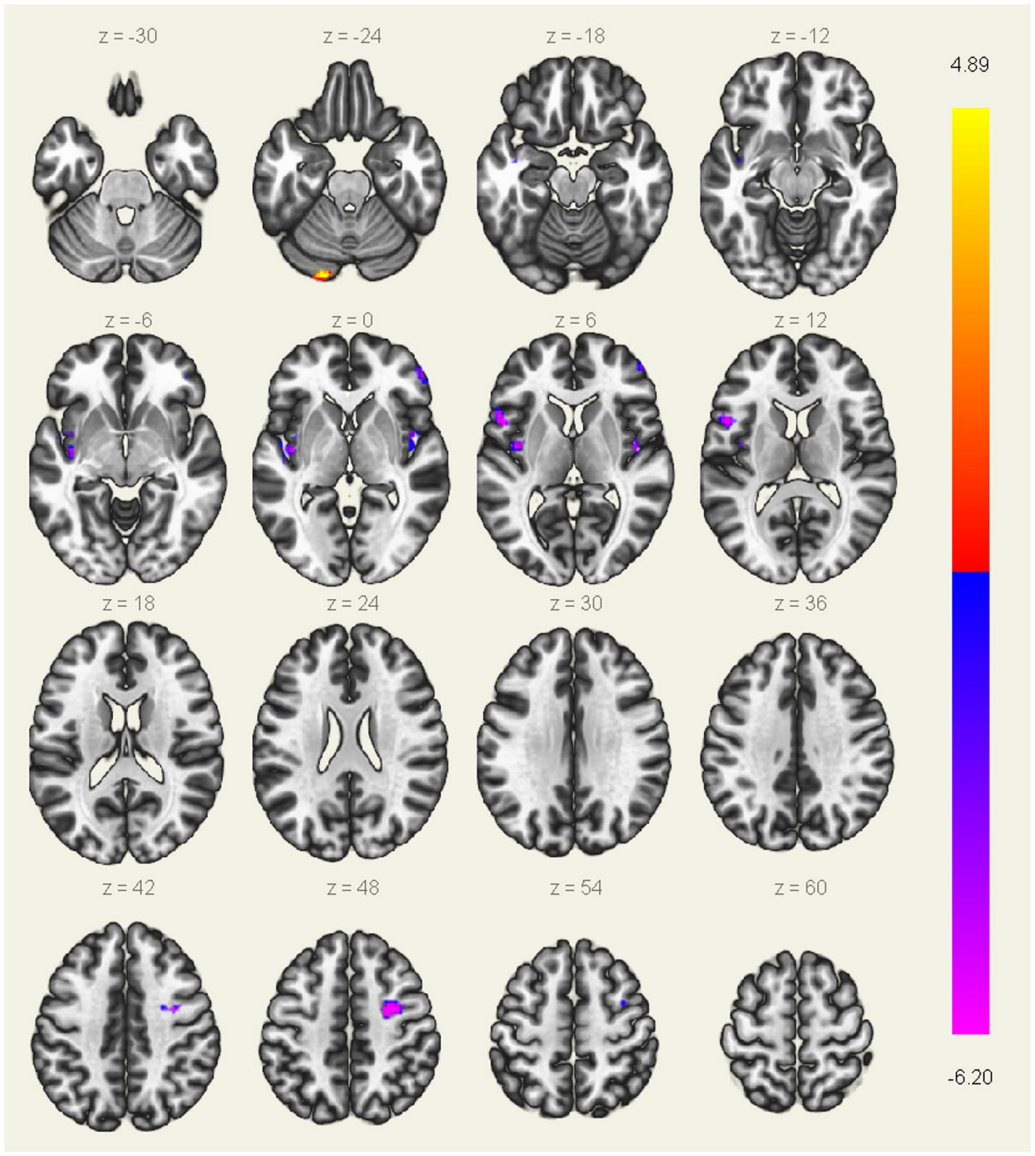
Brain regions with significant differences in ALFF between patients with AUVP and HC. Threshold was set at voxel-level threshold (*p* < 0.001) and cluster-level threshold (*p* < 0.05, FDR corrected, two-tailed). The blue and purple regions represent the brain regions where the ALFF value of acute unilateral vestibulopathy (AUVP) patients is significantly decreased compared with the healthy controls (HC), and the red and yellow regions refer to the brain areas where the ALFF value of AUVP patients is significantly increased. The left side of the picture is the left side of the human brain. FDR, False discovery rate; ALFF, Amplitude of low-frequency fluctuation.

As showing in Table 2 and Figure 1, compared with HC, patients with AUVP showed lower ALFF in brain regions of bilateral insular, right PreCG, left IFG and right MFG, as well as higher ALFF in left CAL (threshold was set at voxel-level threshold (*p* < 0.001) and cluster-level threshold (*p* < 0.05), FDR corrected, two-tailed).

**Table 2:** Brain regions with significant differences in ALFF between patients with AUVP and HC.

Regions	Cluster size	Peak MNI coordinates (x, y, z)	*t*-value	AAL	BA
L-insular	105	−38 −07 09	−6.20	Insula_L	13
R-PreCG	77	27 –03 45	−6.01	Precentral_R	6
L-IFG	65	−51 12 09	−5.89	Frontal_Inf_Oper_L	44
R-insular	38	45 –09 03	−5.23	Insula_R	13
R-MFG	36	51 48 03	−4.94	Frontal_Mid_R	46
L-CAL	34	−20 −87 −24	4.89	Cerebellum crus1_L	18

Threshold was set at voxel-level threshold (*p* < 0.001) and cluster-level threshold (*p* < 0.05, FDR corrected, two-tailed). FDR, False discovery rate; ALFF, Amplitude of low-frequency fluctuation; AUVP, Acute unilateral vestibulopathy; HC, Healthy controls; MNI, Montreal neurological institute; AAL, Anatomical automatic labeling; BA, Brodmann area; L, Left; R, Right; PreCG, Precentral gyrus; IFG, Inferior frontal gyrus; MFG, Middle frontal gyrus; CAL, Cerebellar anterior lobe.

### FC differences between groups

**Figure 2:**
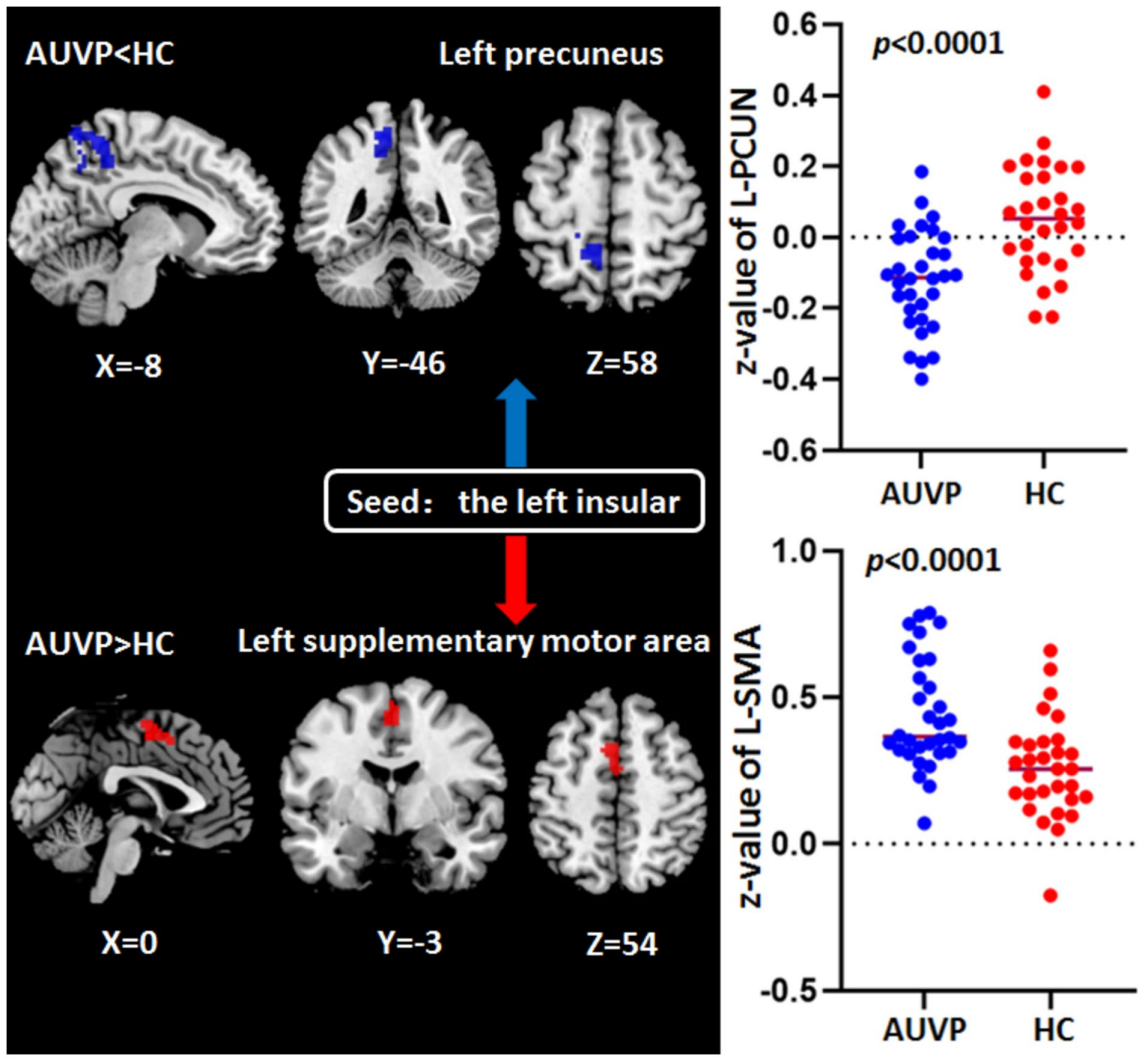
Abnormal functional connectivity of left insular in patients with acute unilateral vestibulopathy (AUVP) compared with healthy controls (HC). Threshold was set at voxel-level threshold (*p* < 0.001) and cluster-level threshold (*p* < 0.05, FDR corrected, two-tailed). FDR, False discovery rate; L, Left; PCUN, Precuneus; SMA, Supplementary motor area.

The FC differences between the two groups were displayed in Table 3 and Figure 2. Compared with HC, patients with AUVP showed decreased FC between the left insular and the left precuneus, as well as increased FC between the left supplementary motor area (SMA) and the left insular (voxel-level threshold *p* < 0.001; cluster-level threshold *p* < 0.05, FDR corrected, two-tailed).

**Table 3:** Abnormal functional connectivity of left insular in AUVP patients compared with HC.

Regions	Cluster size	Peak MNI coordinates (x, y, z)	*t*-value	AAL	BA
L-PCUN	188	−9 −72 36	−5.7085	Precuneus_L	7
L-SMA	73	0 –3 54	4.5662	Supp_Motor_Area_L	6

Threshold was set at voxel-level threshold (*p* < 0.001) and cluster-level threshold (*p* < 0.05, FDR corrected, two-tailed). FDR, False discovery rate; AUVP, Acute unilateral vestibulopathy; HC, Healthy controls; MNI, Montreal neurological institute; AAL, Anatomical automatic labeling; BA, Brodmann area; L, Left; PCUN, Precuneus; SMA, Supplementary motor area.

### Correlation results

**Figure 3:**
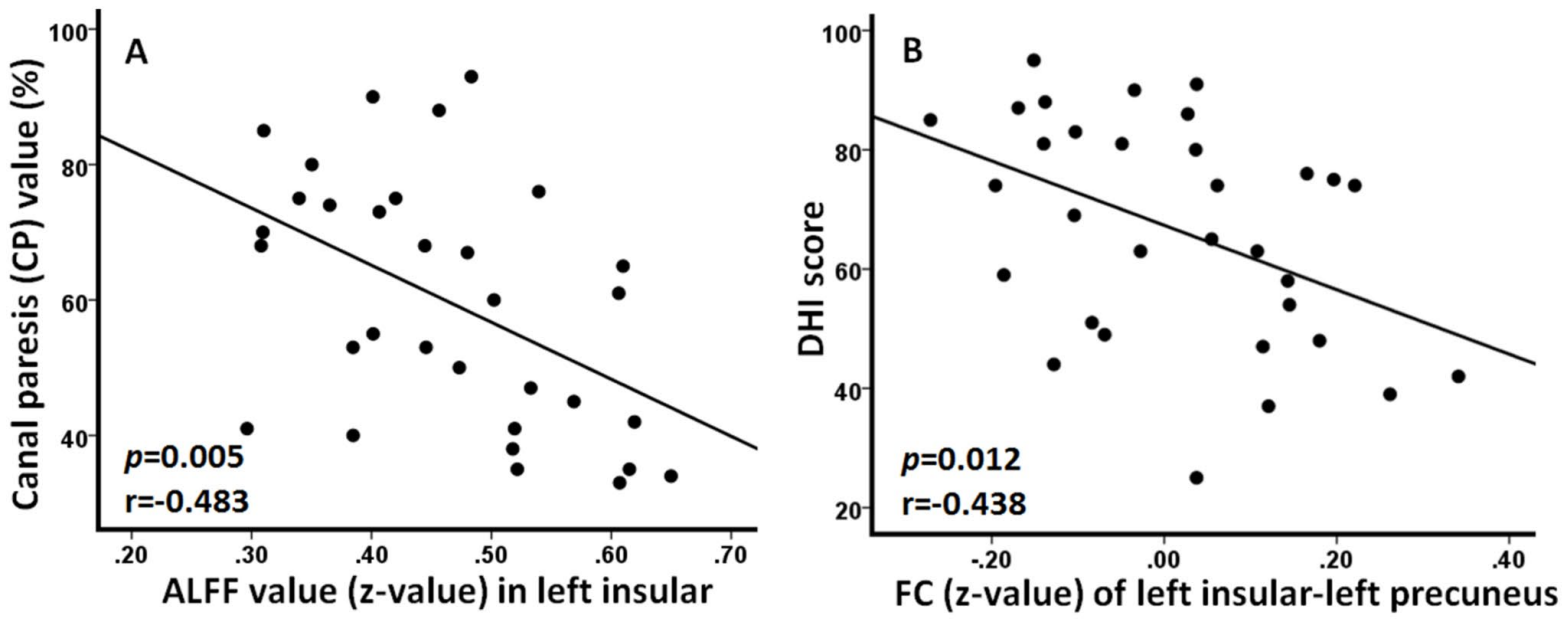
Significant correlations between neuroimaging changes and clinical features in patients with acute unilateral vestibulopathy. **(A)** Amplitude of low-frequency fluctuation (ALFF) value in left insular was negatively correlated with the canal paresis value (*p* = 0.005, *r* = −0.483). **(B)** Functional connectivity (FC) between left insular and left precuneus was negatively correlated with dizziness handicap inventory (DHI) score (*p* = 0.012, *r* = −0.438).

Results of correlation analysis showed that ALFF value (z-value) in the left insular was negatively correlated with the CP value (*p* = 0.005, *r* = −0.483) and FC (z-value) between left insular and left precuneus was negatively correlated with DHI score (*p* = 0.012, *r* = −0.438) in patients with AUVP (Figure 3).

## Discussion

Using methods of ALFF and FC, the current study investigated the changes of brain functional activity and connectivity in patients with AUVP during acute state. We found that compared with HC, the resting-state brain functional activity and connectivity of AUVP patients were altered, and these alterations were closely related to certain clinical characteristics of the patients.

### Altered ALFF in AUVP patients

Compared with HC, the ALFF values of bilateral insula in AUVP patients were significantly lower, suggesting a decline in functional activity of the bilateral insula. Studies have demonstrated that the parieto-insular vestibular cortex (PIVC) is the primary vestibular cortex of human, in which the insula is an important part and plays a crucial role in the processing of vestibular information (16–19). The insular cortex was significantly activated during vestibular stimulation (9, 20). A previous paper suggested that vestibular dysfunction in patients with AUVP is not limited to the peripheral vestibular apparatus and vestibular nucleus, but also involves the central vestibular cortex (21). It has also been reported that patients with AUVP exhibit abnormal cortical activation, characterized by alterations in local glucose metabolism within the insular vestibular cortex on the lesion side. These changes reflect variations in the functional activity of the insular cortex during the resting state (7). A VBM study found that the GMV of the multi-sensory vestibular cortex in patients with chronic AUVP was significantly increased compared to healthy controls, particularly in the insula. This finding suggests a structural compensation in the insular cortical region among chronic AUVP patients (13). Alterations in insular cortical functional activity were also seen in patients with other vestibular disorders, such as persistent postural-perceptual dizziness (PPPD) and vestibular migraine (22, 23). In our study, patients with AUVP exhibited abnormal bilateral insular cortical functional activity, indicating decreased central vestibular processing during the acute stage. Additionally, we found that the functional activity in the left insula was correlated with the CP value of the AUVP patients. Therefore, we propose that the altered functional activity in the insular cortex may be closely associated with the vertigo symptoms experienced by AUVP patients.

We also found decreased ALFF value in the right MFG in patients with AUVP. The MFG receives vestibular information and is believed to be the origin of the direct fibers of the vestibular nucleus (24). Task-state fMRI studies have demonstrated that the MFG is significantly activated in response to electrical or caloric stimulation, suggesting that the MFG plays a crucial role in the vestibular cortex circuit (25, 26). Consequently, the decreased functional activity observed in the right MFG in our study may indicate a reduction in vestibular information processing in patients with AUVP.

The PreCG is the primary motor cortex of the human body, receiving information from the cerebellum and basal ganglia via the sensory area of the parietal lobe and the projection from the thalamo-cortex. It plays a crucial role in motion control and gait balance. Previous functional neuroimaging studies have found that caloric or electrical stimulation of the peripheral vestibular area can generate symptoms of vertigo. At the same time, the PreCG was significantly activated (27, 28). It has been reported that patients with PPPD showed reduced GMV in the PreCG (29). In the present study, we observed a significant reduction in the functional activity of the right PreCG in patients with AUVP. We hypothesize that this decrease in functional activity may be associated with the directional imbalance of bilateral vestibular tension resulting from acute unilateral vestibular injury, which in turn contributes to balance disorders and instability in AUVP patients.

We also observed decreased ALFF value in the left IFG in patients with AUVP. The left IFG (BA44/45/47) was proved by a meta-analysis to be involved in motor control (30). It was also suggested that the left IFG participated in motion perception and in the evaluation of action intentions (30). Therefore, we believed that the decreased functional activity in left IFG was also related to the impaired motion control in patients with AUVP.

The projection of the vestibular nucleus extends into the cerebellum, which plays an important role in maintaining balance and coordination during goal-oriented movement (31, 32). The cerebellum is closely related to vertigo, and stroke accompanied by cerebellar injury is one of the most common causes of vascular vertigo (33). The CAL (cerebellum crus1 in our study) has been reported to be associated with spatial cognition and plays an important role in spatial navigation and positioning (34, 35). The current study identified an increased ALFF value in the left CAL, which may reflect impairments in balance and spatial cognition in AUVP patients.

### Altered FC in AUVP patients

In seed-based FC analysis, we found decreased FC between left insular and left precuneus in AUVP patients. The precuneus is a crucial part of posterior default mode network (pDMN), which is believed to be involved in attention monitoring and self-centered cognition (36, 37). A psychologist suggested that the precuneus might be involved in human emotional processing (38). Although vertigo is often accompanied by anxiety or depression, the subjects included in our study showed no obvious moderate–severe emotional disorders. Thus, the altered functional activity in bilateral precuneus might have less to do with emotion. A previous fMRI study indicated that the preceneus was engaged in visual information processing as they observed increased precuneus activation during optokinetic stimulation (39). Additionally, it has been reported that electrical stimulation of the precuneus can induce symptoms of vertigo, indicating that the precuneus may play a crucial role in processing vestibular information (40, 41). We propose that the precuneus is part of the multi-sensory vestibular cortex, and the decreased functional connectivity between the insula and precuneus may reflect abnormal functional activity within the multi-sensory vestibular cortex in patients with AUVP.

Furthermore, increased FC between the left insular and the left SMA was observed in AUVP patients. The SMA was reported to be involved in self-initiated and triggered movements (42, 43). The enhanced FC between the contralesional insula and SMA is likely to be a result of relative contralesional activation leading to increased transmission of vestibular information from the contralesional side to posture-regulating areas.

### Limitations

There are some limitations in the present study. Firstly, the sample size was relatively small, so this study was not representative of the entire AUVP population. Future studies should consider larger, more diverse patient populations to enhance the generalizability of the results. Secondly, lesion side and the handedness of the subjects had a complex interplay on brain functional changes after AUVP (8). To control for this, only right-handed subjects with right sided AUVP were included in this study. However, we did not compare the differences in brain functional changes between patients with left and right lesions. The third potential limitation was that we did not follow up patients with AUVP to observe changes in brain functional activity after 3 months to further verify the results of this study. Finally, future studies should integrate additional imaging analysis techniques to further investigate the brain functional changes in patients with AUVP. Examples of such methods include independent component analysis (ICA), functional network connectivity (FNC), and dynamic functional network connectivity (dFNC).

## Conclusion

Patients with AUVP during acute period showed altered functional activity and connectivity in brain regions mainly involved in motor control and vestibular information processing. These changes in brain functional activity and connectivity were potentially attributed to decreased vestibular input resulting from unilateral peripheral vestibular impairment. Our findings offer novel insights into the brain functional changes following acute unilateral peripheral vestibular impairment.

## Data Availability

The raw data supporting the conclusions of this article will be made available by the authors, without undue reservation.

## Footnotes

^1^ http://www.Nitrc.org/projects/conn

^2^ http://www.fil.ion.ucl.ac.uk/spm/software/spm12
